# Representation in the UK MS Register: A Comparison of Nationwide Ethnicity and Register Data

**DOI:** 10.23889/ijpds.v9i5.2875

**Published:** 2024-09-18

**Authors:** Rod Middleton, Elaine Craig

**Affiliations:** 1 Swansea University

## Background

The United Kingdom MS Register (UKMSR) collects longitudinal data on people with Multiple Sclerosis (pwMS) from clinical sources and as Patient Reported Outcomes (PROs) via a web portal. People from non-white ethnicities are often poorly represented in research and clinical trials; the UKMSR aims to be representative of people with MS (pwMS) across the UK.

## Aim

To compare ethnic representation in the UKMSR to data from the Office for National Statistics (ONS).

## Method

Ethnicity data was taken from the UKMSR as a ‘snapshot’ in 2024. Comparative data from ONS in England and Wales was gathered. Due to low numbers in the UKMSR of some ethnicities and areas, participants were grouped into ‘White’ and ‘All Other Ethnicities’. Using Chi-Sq tests, we compared ethnicities across ten regions of ONS 2021 Census data.

## Results

Ten regions were compared (nine in England, Wales as one). Analysis of both web-reported (n = 18648) and clinical (n = 5456) ethnicity found UKMSR diversity to be significantly lower than national statistics (p < .05), with the exception of the clinical Register in Wales (numbers were insufficient to test) and clinical Register in the English West Midlands. The clinical Register was more representative in 7/10 regions than the web Register.

## Discussion

The clinical Register, via NHS hospital sites reaches a more diverse audience than the patient-reported web Register; further work is needed in reaching a broader audience of pwMS, as well as harmonising discrepancies between the clinical and web Register populations.

